# Combined Day–Night Heat Damage in Soybean Is Driven by Nighttime Warming

**DOI:** 10.1111/ppl.71100

**Published:** 2026-10-18

**Authors:** Gabriel Ferraz Guimarães, Jéssica de Jesus Santos, Rafael Vasconcelos Ribeiro, Milton Costa Lima Neto

**Affiliations:** ^1^ Institute of Biosciences, Department of Biological and Environmental Sciences, Laboratory of Plant Metabolism São Paulo State University (UNESP) São Vicente SP Brazil; ^2^ Institute of Biology (IB), Department of Plant Biology, Laboratory of Crop Physiology State University of Campinas (UNICAMP) Campinas SP Brazil

**Keywords:** asymmetric warming, *Glycine max*, nocturnal heat, oxidative stress, stomatal limitation

## Abstract

Asymmetric warming, characterized by faster increases in nighttime than daytime temperatures, is an emerging threat to crop performance. However, the interactive physiological effects of day versus night heat remain insufficiently resolved in soybean ( *Glycine max* L. Merr.). Here, we evaluated soybean responses to 4 day/night thermal regimes (28°C/22°C, 40°C/22°C, 28°C/28°C, and 40°C/28°C) to test whether nighttime warming is the dominant driver of stress disruption. Isolated daytime heat (40°C/22°C) enhanced photosynthetic performance and maintained biomass, indicating short‐term functional plasticity. In contrast, treatments including elevated nighttime temperature impaired pre‐dawn rehydration, reduced leaf water status, and intensified membrane damage, with the strongest effects under combined heat (40°C/28°C). Combined stress also caused severe stomatal limitation of CO_2_ assimilation, depletion of soluble sugars, accumulation of proline and glycine betaine, and strong activation of antioxidant enzymes accompanied by high H_2_O_2_ and TBARS levels, indicating that oxidative load exceeded detoxification capacity. Multivariate integration reinforced this pattern by clearly separating combined heat from all other treatments and placing nighttime warming as the central axis of physiological disruption. Together, our findings demonstrate that restricted nocturnal recovery is a key mechanism underlying synergistic damage under sustained day–night heat, highlighting nighttime metabolism and overnight hydraulic recovery as priority targets for improving soybean heat resilience.

## 1 Introduction

Soybean ( *Glycine max* L. Merr.) stands as a cornerstone of global agriculture, providing a substantial portion of the world's protein and vegetable oil supply essential for both human and animal consumption. However, its productivity is increasingly threatened by the impacts of global climate change, particularly rising air temperatures (Thenveettil et al. 2024). Projections indicate that this warming will continue, posing a considerable threat to soybean yields worldwide (Yang et al. 2023). A critical aspect of this trend is the disproportionate rise in nighttime temperatures compared with daytime warming, a phenomenon known as asymmetric warming that introduces unique challenges for crops (Mall et al. 2021). Research has consistently shown that elevated temperatures during critical developmental stages like seed filling are detrimental, leading to significant yield losses and compromised seed quality (Giménez et al. 2025). Recent studies have demonstrated that high nighttime temperature causes a 4.6% yield decline per °C in soybean, while temperature deviations from optimal levels during flowering and seed‐filling stages can result in an 83% yield reduction (Thenveettil et al. 2024; Li et al. 2024). Given these pressing concerns, a nuanced understanding of the differential effects of elevated day versus night temperatures on soybean physiology and metabolism is crucial for ensuring future food security.

The physiological impacts of elevated temperatures on soybean are multifaceted and depend significantly on whether the stress occurs during the day or night. Elevated daytime temperatures directly impair the photosynthetic machinery. Temperatures exceeding the optimum threshold for soybean photosynthesis destabilize thylakoid membranes and damage photosystem II (PSII), reducing ATP and NADPH synthesis and decreasing the activation and carboxylation efficiency of Rubisco (Song et al. 2014; Petrova et al. 2018; Herritt et al. 2020). These conditions also increase photorespiration (Busch 2020). As leaf temperature rises, the CO_2_/O_2_ solubility ratio in the chloroplast stroma declines and the specificity of Rubisco for CO_2_ decreases, thereby favoring Rubisco oxygenation and enhancing photorespiratory flux (Busch 2020; Slattery and Ort 2019). The resulting diversion of energy and reducing power into the recycling of 2‐phosphoglycolate further lowers the efficiency of carbon gain in soybean leaves and exacerbates the negative impact of heat on photosynthesis (Busch 2020; Slattery and Ort 2019).

While a protective mechanism, stomatal closure to conserve water under high evaporative demand also restricts CO_2_ uptake, further limiting photosynthesis and leading to reduced pod and seed numbers (Yang et al. 2023). In contrast, elevated night temperatures introduce a different set of challenges, primarily centered on carbon metabolism (Yang et al. 2023). Increased nighttime respiration consumes a substantial portion of the carbohydrates produced during the day, leading to a state described as “carbon hunger” (Thenveettil et al. 2024). This imbalance disrupts the partitioning of sugars to developing seeds and can downregulate the expression of key genes involved in sucrose synthesis and transport. While some studies report a transient initial increase in photosynthetic rates in response to high nighttime temperature, this effect is typically followed by a gradual decline, and the prolonged metabolic strain ultimately contributes to significant yield losses by reducing seed size and weight (Yang et al. 2023; Thenveettil et al. 2024).

In soybean, net photosynthesis under saturating light typically exhibits an optimal temperature range of approximately 25°C–30°C, although the exact optimum can vary among cultivars and with growth temperature (Thenveettil et al. 2024). Above this range, progressive Rubisco deactivation together with constraints on chloroplast electron transport increasingly co‐limit CO_2_ assimilation, resulting in a steep decline in photosynthetic rates (Moore et al. 2021). Although a single precise critical temperature for photochemical damage has not been firmly established for soybean, evidence from leaves and cell suspension cultures indicates that temperatures around 41°C–45°C already cause substantial but partly reversible PSII inhibition, whereas exposure above 45°C leads to irreversible injury to the PSII reaction center, compromising both the donor and acceptor sides of PSII (Yamane et al. 1997; Zheng et al. 2009). Consistently, in common bean, the critical temperature for photochemical damage was estimated in the 40°C–45°C range based on an abrupt decline in the apparent rate of PSII electron transport (ETR), suggesting that grain legumes share a relatively narrow thermal window before photochemical injury is triggered (Ribeiro et al. 2015).

While a substantial body of research has documented the effects of either daytime or nighttime heat stress (Zheng et al. 2009; Giménez et al. 2025; Mall et al. 2021), a comprehensive understanding of their comparative and interactive impacts, particularly within a single experimental framework, remains less clear. Many studies focus on one aspect of the diurnal cycle or on constant high temperatures, leaving a critical gap in knowledge regarding how soybean plants respond to the distinct physiological challenges posed by isolated versus combined high day and high night temperatures. Recent research has highlighted the importance of asymmetric diurnal temperature changes, with studies showing that the warming trend exhibits an asymmetrical increase due to more rapid increases in nighttime temperatures relative to daytime temperatures (Mall et al. 2021). However, most current understanding is based on studies that examine either daytime or nighttime effects in isolation, potentially missing important interactive effects that occur when both stresses are present simultaneously.

To accurately simulate these distinct thermal limits within a controlled experimental setup, the selection of absolute treatment temperatures must target the specific physiological thresholds of injury rather than strictly duplicating climatological warming rates. While net photosynthesis in soybean remains optimal up to 30°C, pushing the daytime machinery to 40°C is necessary to challenge photochemical capacity and induce significant thermal stress without causing immediate mortality (Moore et al. 2021; Ribeiro et al. 2015). Conversely, because the baseline for overnight recovery sits around 22°C, a nighttime elevation to 28°C represents a critical threshold that significantly intensifies respiratory strain, impairs hydraulic rehydration, and triggers carbon limitations (Lin et al. 2021; Yang et al. 2023). This experimental setup therefore establishes a realistic yet challenging gradient to investigate how these independent diurnal forces interact during the transition from vegetative to early reproductive growth.

Therefore, the objective of this study was to dissect the differential and interactive effects of elevated day versus night temperatures on the growth, water status, photosynthetic performance, and key biochemical stress responses of soybean plants. We tested the central hypothesis that elevated night temperature is more detrimental to soybean physiology than elevated day temperature. We posited that this is due to the impairment of essential overnight metabolic repair and the acceleration of respiratory carbon loss, which destabilizes the plant's overall carbon balance. The enhanced nocturnal respiration under elevated night temperatures leads to excessive consumption of photosynthetic products, creating carbon limitation for seed development. Furthermore, elevated night temperatures disrupt the normal circadian regulation of metabolic processes and compromise the plant's antioxidative defense system. Consequently, we predicted that the combined stress of high day and night temperatures would exert a synergistic, rather than merely additive, negative impact, leading to a severe collapse in photosynthetic capacity and an overwhelming of the antioxidative defense system.

## 2 Materials and Methods

### 2.1 Plant Material, Growth Conditions, and Experimental Design

Seeds of soybean ( *Glycine max* L. Merr.) cv. BRS 546 (EMBRAPA, Brazil) were surface sterilized by immersion in 1% sodium hypochlorite for 2 min, followed by thorough rinsing with tap water. The seeds were then soaked in distilled water for 3 h before sowing. Sowing was done in 5 L plastic pots filled with a 1:1 (v/v) mixture of washed sand and a commercial potting substrate. The pots were maintained in growth chambers (SOLIDSTEEL SSCCU630L‐110, São Paulo, SP Brazil) under the following controlled conditions: 12‐h photoperiod with a photosynthetic photon flux density (PPFD) of 400 μmol m^−2^ s^−1^, a day/night temperature regime of 28°C/22°C, and 65% ± 5% relative air humidity. Plants were watered daily to the maximum water holding capacity of the substrate using distilled water. To compensate for the higher evaporative demand in the elevated temperature chambers, the irrigation volume was adjusted dynamically based on daily pot weight assessments, ensuring that substrate moisture remained nonlimiting and statistically uniform across all treatments. Additionally, plants were fertilized every 15 days with a half strength Hoagland and Arnon (1950) nutrient solution.

At the V2 stage, the plants were randomly assigned to one of four temperature treatments (day/night), conducted in separate growth chambers: (i) Control (C, 28°C/22°C); (ii) High night temperature (HN, 28°C/28°C); (iii) High day temperature (HD, 40°C/22°C); and (iv) Combined high day and night temperature (HDN, 40°C/28°C). The thermal regimes were delivered using a rectangular diurnal profile without gradual ramping periods. The transition between daytime and nighttime target temperatures was synchronized with the automated switching of the 12 h photoperiod light banks, with the internal atmosphere of each independent chamber stabilizing at the designated treatment temperature within 40 to 45 min following each transitional phase. Each treatment consisted of five replicate pots, with one plant per pot constituting a single replicate. The treatments were maintained for 20 days, after which all plants were harvested for physiological and biochemical analyses. This duration was chosen as the experimental endpoint because it corresponded to the time point when plants in the most severe treatment (40°C/28°C) began to exhibit clear and irreversible visual symptoms of stress, such as sustained turgor loss and chlorosis of older leaves.

### 2.2 Plant Growth and Water Status, Membrane Integrity, and Photosynthetic Pigments

At the end of the 20‐day treatment period, plants were harvested for analysis. The aboveground biomass (stems + leaves) of each plant was excised, and its fresh mass (FM) was immediately recorded using a precision balance. Leaf water potential (Ψ) was measured on fully expanded leaves using a pressure chamber (Model 600, PMS Instrument Co.), following Scholander et al. (1965). Measurements were taken at two time points: predawn (Ψ_*PD*_), between 04:00 and 05:00, and midday (Ψ_*MD*_), between 12:00 and 13:00. Leaf relative water content (RWC) was determined according to Cenciareli et al. (2025). Briefly, fully expanded leaves were excised, their fresh mass (FM) was promptly recorded, and they were then hydrated by floating on deionized water in darkness for 12 h to obtain the turgid mass (TM). Finally, samples were oven‐dried at 65°C until a constant dry mass (DM) was achieved. RWC was calculated as: RWC (%) = [(FM−DM)/(TM−DM)] × 100.

Membrane damage (MD) was assessed by measuring electrolyte leakage, adapted from Lima Neto et al. (2017). Leaf discs of a known area were thoroughly washed with deionized water and placed in test tubes containing 10 mL of deionized water. The tubes were incubated on a shaker at room temperature for 12 h, after which the initial electrical conductivity (L1) of the solution was measured using a conductivity meter. To release electrolytes, the samples were then boiled at 95°C for 20 min. After cooling to room temperature, the final electrical conductivity (L2) was measured. The percentage of membrane damage was calculated as: MD (%) = (L1/L2) × 100. Photosynthetic pigments were extracted from fresh leaf tissue (100 mg) with 80% (v/v) cold acetone. The homogenate was centrifuged, and the absorbance of the supernatant was measured at 663, 645, and 470 nm using a spectrophotometer (GENESYS 10S UV–Vis, Thermo Fisher). The leaf concentrations of chlorophyll *a*, chlorophyll *b*, and total carotenoids were calculated using the equations established by Lichtenthaler (1987).

### 2.3 Leaf Gas Exchange and Chlorophyll *a* Fluorescence

Leaf gas exchange and chlorophyll *a* fluorescence parameters were measured simultaneously between 09:00 and 11:00 on the second fully expanded leaf of each plant after 20 days of treatment using a portable open flow gas exchange system (GFS 3000 FL, Heinz Walz GmbH). The leaf chamber parameters were carefully regulated according to established protocols (Busch et al. 2024). The cuvette block temperature was adjusted dynamically to match the respective daytime growth temperature of each treatment group, being maintained at either 28°C or 40°C. To prevent water condensation inside the leaf chamber and avoid artifactual atmospheric constraints at elevated temperatures, the humidity of the incoming air stream was tightly regulated to maintain the leaf to air vapor pressure deficit (VPD) strictly within a stable range between 1.0 and 1.7 kPa. All measurements were performed at an ambient CO_2_ concentration of 400 μmol mol^−1^, a photosynthetic photon flux density (PPFD) of 500 μmol m^−2^ s^−1^ containing 10% blue light. This slightly higher measurement PPFD relative to the growth‐chamber condition (400 μmol m^−2^ s^−1^) was a deliberate choice, since our growth chambers could not reach fully saturating light intensities; even at 500 μmol m^−2^ s^−1^, irradiance remained below the light‐saturation point for soybean leaves, allowing treatment differences to be resolved without saturating the photosynthetic apparatus. Gas exchange variables, including net photosynthetic rate (*A*), stomatal conductance (*gs*), transpiration rate (*E*), and intercellular CO_2_ concentration (*Ci*), were recorded after achieving stable steady state stabilization, while leaf temperature was tracked continuously via the internal chamber thermocouple to monitor transpirational cooling dynamics.

Immediately thereafter, chlorophyll *a* fluorescence was assessed on the same light‐adapted leaf. A saturating light pulse (8000 μmol m^−2^ s^−1^ for 0.7 s) was applied to determine maximum fluorescence ($F_{m}^{\prime }$), allowing calculation of the effective quantum efficiency of PSII [Y(II)]. The electron transport rate (ETR) was calculated as ETR = Y(II) × PPFD × 0.84 × 0.5, where 0.84 was assumed as leaf absorptance and 0.5 assumes equal distribution of absorbed energy between PSII and PSI (Murchie and Lawson 2013). Nonphotochemical quenching (NPQ) was calculated as $\left(F_{m}-F_{m}^{\prime }\right)/F_{m}^{\prime }$. To evaluate the maximum quantum efficiency of PSII (*F*_*v*_ */F*_*m*_), the same leaves were dark‐adapted for 30 min before measuring the minimum (*F*_*o*_) and maximum (*F*_*m*_) fluorescence yields; *F*_*v*_ */F*_*m*_ values were then obtained as (*F*_*m*_−*F*_*o*_)/*F*_*m*_ (Murchie and Lawson 2013).

### 2.4 Osmolytes and Soluble Proteins

For all biochemical analyses, fresh leaf samples were collected at harvest (after 20 days of treatment), immediately frozen in liquid nitrogen, and stored at −80°C until analysis. Total soluble sugars (TSS) were quantified according to the anthrone method (Yemm and Willis 1954). Briefly, approximately 100 mg of frozen leaf tissue was homogenized in 80% ethanol and incubated in a water bath at 80°C for 30 min. After centrifugation, the supernatant was mixed with a solution of 0.2% anthrone in concentrated sulfuric acid. The reaction was developed by incubating the mixture at 95°C for 10 min, followed by rapid cooling to room temperature in an ice bath. Absorbance was measured at 620 nm with a spectrophotometer (GENESYS 10S UV–Vis, Thermo Fisher). TSS content was determined from a standard calibration curve constructed with D‐glucose.

Proline concentration was measured using the acid ninhydrin method (Bates et al. 1973). Approximately 100 mg of frozen leaf tissue was homogenized in 3% (w/v) sulfosalicylic acid. The extract was reacted with acid ninhydrin reagent at 100°C for 1 h. After immediate cooling in an ice bath, the chromophore was extracted with toluene and the absorbance of the organic phase was measured at 520 nm. Proline content was calculated based on a standard curve prepared with L‐proline. Glycine betaine (GB) content was assessed according to Grieve and Grattan (1983). Finely powdered leaf material was mixed with 2 N H_2_SO_4_, and an aliquot was reacted with cold potassium iodide‐iodine solution for 16 h at 4°C. Periodide crystals were separated by centrifugation, dissolved in 1,2‐dichloroethane, and the absorbance was measured at 365 nm. GB concentration was determined using a standard curve prepared with known concentrations of glycine betaine.

Total soluble protein (TSP) content was determined using the Bradford assay (Bradford 1976). Approximately 100 mg of frozen leaf tissue was homogenized in potassium phosphate buffer (pH 7.0, 100 mM), containing 1 mM ascorbate and 2 mM EDTA. The homogenate was centrifuged at 12000 *g* for 15 min at 4°C. An aliquot of the supernatant was then reacted with Coomassie Brilliant Blue G‐25 dye reagent, and the absorbance was read at 595 nm after 10 min of incubation. Protein concentration was calculated against a standard curve prepared with bovine serum albumin (BSA).

### 2.5 Oxidative Stress Markers and Antioxidant Enzyme Assays

Lipid peroxidation was quantified by measuring thiobarbituric acid reactive substances (TBARS) following Cakmak and Horst (1991). The crude extract was mixed with 20% trichloroacetic acid (TCA) and 0.5% thiobarbituric acid (TBA), heated at 95°C for 30 min, and rapidly cooled on ice. After centrifugation, absorbance of the supernatant was read at 532 nm and corrected for nonspecific absorbance at 600 nm. TBARS concentration was calculated using an extinction coefficient of 155 mM^−1^ cm^−1^, with the results expressed as malondialdehyde (MDA) equivalents to accurately reflect the index of lipid peroxidation. Hydrogen peroxide (H_2_O_2_) content was determined using the Amplex Red reagent (10‐acetyl‐3,7‐dihydroxyphenoxazine), as described by Zhou et al. (1997). The reaction mixture contained 10 mM Amplex Red and 10 U horseradish peroxidase in phosphate buffer. Absorbance of the fluorescent product, resorufin, was measured spectrophotometrically at 560 nm, and H_2_O_2_ concentration was calculated against a calibration curve.

For the determination of antioxidant enzyme activities, approximately 200 mg of frozen leaf tissue were homogenized in 1 mL ice cold 100 mM potassium phosphate buffer at pH 7.0, containing 2 mM EDTA and 1 mM ascorbate. The homogenate was centrifuged at 12000 g for 20 min at 4°C, and the resulting supernatant was collected and used directly as the crude enzyme extract. Superoxide dismutase (SOD; EC 1.15.1.1) activity was assayed via inhibition of nitroblue tetrazolium (NBT) photoreduction according to Giannopolitis and Ries (1977). The assay reaction included enzyme extract, 50 mM potassium phosphate buffer (pH 7.8), 13 mM methionine, 75 μM NBT, 0.1 mM EDTA, and 2 μM riboflavin. Samples were illuminated for 6 min, and absorbance was measured at 560 nm. One unit of SOD was defined as the amount of enzyme required to inhibit the NBT reduction rate by 50%. Catalase (CAT; EC 1.11.1.6) activity was measured by directly following H_2_O_2_ decomposition at 240 nm, as described by Havir and McHale (1987). The reaction was initiated by addition of enzyme extract to 50 mM potassium phosphate buffer (pH 7.0) containing 20 mM H_2_O_2_. Enzymatic activity was computed from the rate of absorbance decrease using the extinction coefficient for H_2_O_2_ (36 M^−1^ cm^−1^). Ascorbate peroxidase (APX; EC 1.11.1.11) activity was determined by monitoring the oxidation of ascorbate at 290 nm (Nakano and Asada 1981). The assay mixture consisted of 50 mM potassium phosphate buffer (pH 7.0), 0.5 mM ascorbate, 0.1 mM EDTA, and enzyme extract. The reaction was started by adding 0.1 mM H_2_O_2_, and APX activity was calculated using the extinction coefficient for ascorbate (2.8 mM^−1^ cm^−1^). All enzyme activities were normalized to protein concentration, which was determined by the Bradford method (Bradford 1976) as detailed in Section 2.4.

### 2.6 Statistical Analysis

The experiment was structured as a completely randomized design, with five biological replicates per treatment. All quantitative data were first evaluated for normality (Shapiro–Wilk test) and homogeneity of variances (Levene's test). Data sets meeting these assumptions were subsequently analyzed using one‐way analysis of variance (ANOVA), followed by Tukey's honestly significant difference (HSD) post hoc test for pairwise mean comparisons (significance threshold set at *p* ≤ 0.05). For integrative interpretation of the plant responses, Principal Component Analysis (PCA) was performed on the entire data matrix. Prior to PCA, variables were autoscaled (mean‐centered and scaled to unit variance). Statistical analyses were conducted using R software (R Core Team 2024), with PCA performed via the FactoMineR package and biplots generated using factoextra.

## 3 Results

### 3.1 Plant Growth, Water Status, and Photosynthetic Pigments

High temperature stress caused marked physiological changes in soybean plants, with the most severe effects observed under the combined high day/night temperature (40°C/28°C). As shown in Table 1, shoot biomass was significantly reduced only under combined heat stress, whereas isolated day (40°C/22°C) or night (28°C/28°C) stress alone did not alter shoot biomass compared to the control. All heat treatments decreased leaf relative water content (RWC) compared to the control, with the lowest RWC recorded under combined heat stress, indicating severe dehydration. Membrane damage (MD) also increased with higher day or night temperature and was highest under combined heat stress, with an approximately threefold increase relative to the control (Table 1). Photosynthetic pigments were differentially affected by heat stress. The maximal chlorophyll *a* (Chl*a*) content occurred under high day temperature (40°C/22°C), whereas combined heat stress caused a drastic depletion in Chl*a* (Table 1). In contrast, chlorophyll *b* (Chl*b*) and carotenoid concentrations peaked under the combined heat stress regime. The lowest Chl*a/b* ratio was found under combined heat stress (Table 1).

**TABLE 1:** Shoot biomass, leaf relative water content (RWC), membrane damage (MD), chlorophylls *a* and *b* (Chl*a*, Chl*b*), chlorophyll *a*/*b* ratio (Chl*a*/Chl*b*), and carotenoids (Car) content in soybean leaves.

Variables	28°C/22°C	28°C/28°C	40°C/22°C	40°C/28°C
Shoot biomass (g)	11.55 ± 0.80a	11.91 ± 0.16a	11.25 ± 0.64a	9.08 ± 0.50b
RWC (%)	81.86 ± 1.35a	70.89 ± 2.33b	74.48 ± 1.43b	60.59 ± 1.55c
MD (%)	18.00 ± 1.58d	47.08 ± 1.91b	39.00 ± 2.45c	54.64 ± 2.38a
Chl*a* (mg g^−1^ FM)	1.19 ± 0.05b	1.21 ± 0.05b	1.45 ± 0.11a	0.69 ± 0.06c
Chl*b* (mg g^−1^ FM)	0.33 ± 0.04b	0.58 ± 0.04a	0.40 ± 0.03b	0.59 ± 0.03a
Chl*a*/Chl*b*	3.60 ± 0.37a	2.09 ± 0.20b	3.66 ± 0.56a	1.17 ± 0.16c
Car (mg g^−1^ FM)	0.33 ± 0.05b	0.40 ± 0.04b	0.36 ± 0.03b	0.54 ± 0.06a

*Note:* Data are represented as the mean ± standard deviation (*n* = 5). Different letters indicate significant differences among treatments according to Tukey's HSD test at *p <* 0.05.

Leaf water potentials were negatively impacted by the heating regimes (Figure 1). At pre‐dawn (Ψ_*PD*_), all heat‐stressed plants exhibited significantly lower water potentials than the control, with the lowest value under combined heat stress. Midday water potentials (Ψ_*MD*_) further decreased across all treatments in response to higher evaporative demand. Control plants reached a Ψ_*MD*_ of −0.41 ± 0.05 MPa, while combined heat‐stressed plants exhibited a sharp decline in Ψ_*MD*_ to −1.18 ± 0.07 MPa (Figure 1).

**FIGURE 1:**
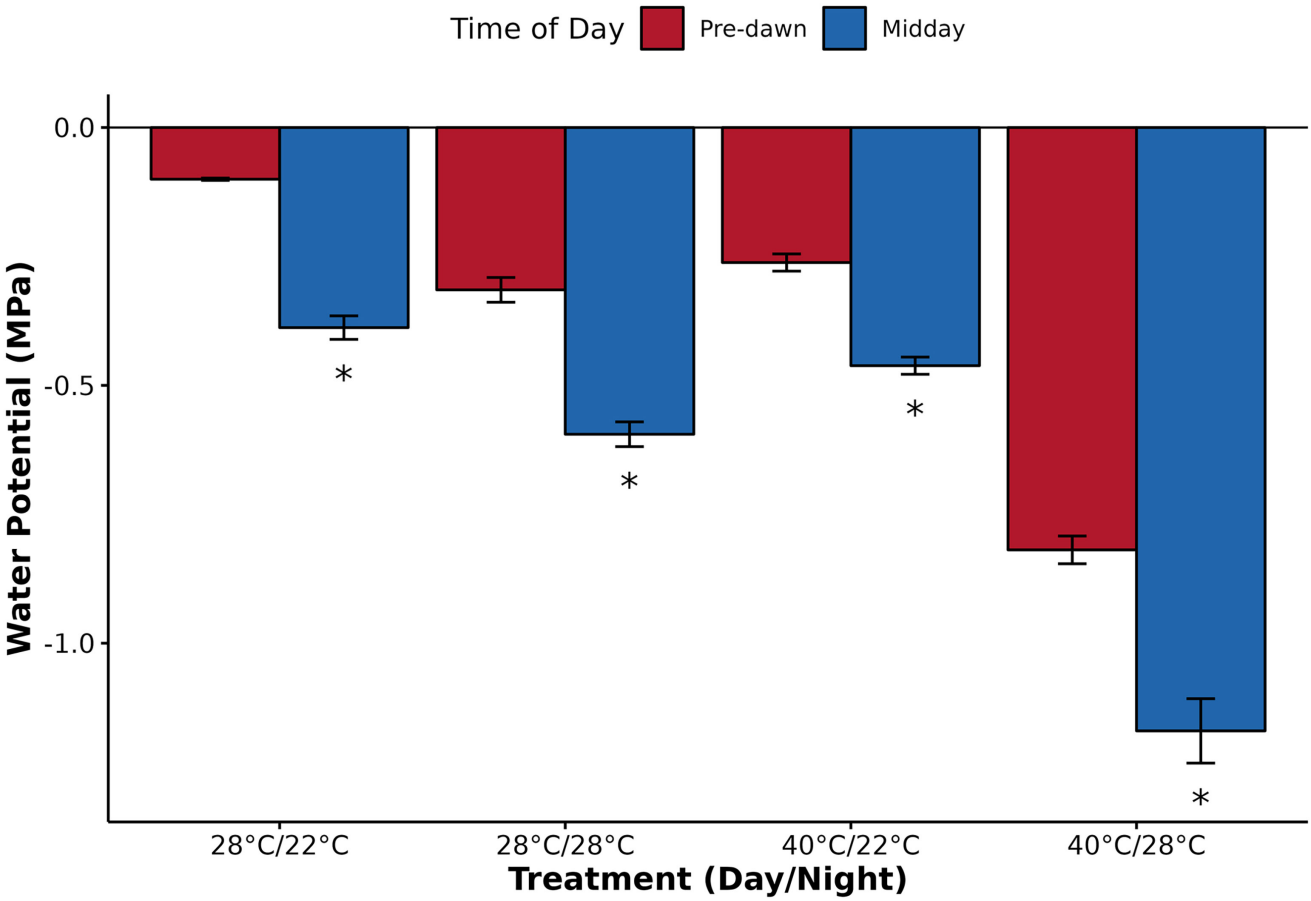
Leaf water potential of soybean plants subjected to different day/night temperature regimes. Measurements were taken at pre‐dawn and midday. Bars represent the mean ± standard deviation (*n* = 5). Different lowercase letters indicate significant differences among treatments within the same measurement period (Pre‐dawn or Midday) according to Tukey's HSD test at *p <* 0.05. Asterisks (*) indicate significant differences between pre‐dawn and midday values within the same treatment according to a Student's *t*‐test at *p <* 0.05.

### 3.2 Leaf Gas Exchange and Chlorophyll *a* Fluorescence

Leaf gas exchange parameters were significantly affected by the temperature regimes (Figure 2). Plants exposed to isolated high day temperatures (40°C/22°C) showed higher net photosynthetic rates (A), stomatal conductance (*g*_*s*_), and transpiration (E) than the control and the other heat treatments. In contrast, the high night treatment (28°C/28°C) and, especially, the combined high day and night treatment (40°C/28°C) led to pronounced reductions in all gas exchange variables. This decline was accompanied by lower intercellular CO_2_ concentration (*C*_*i*_), indicating that photosynthetic inhibition under combined heat stress was primarily driven by stomatal limitation (Figure 2D).

**FIGURE 2:**
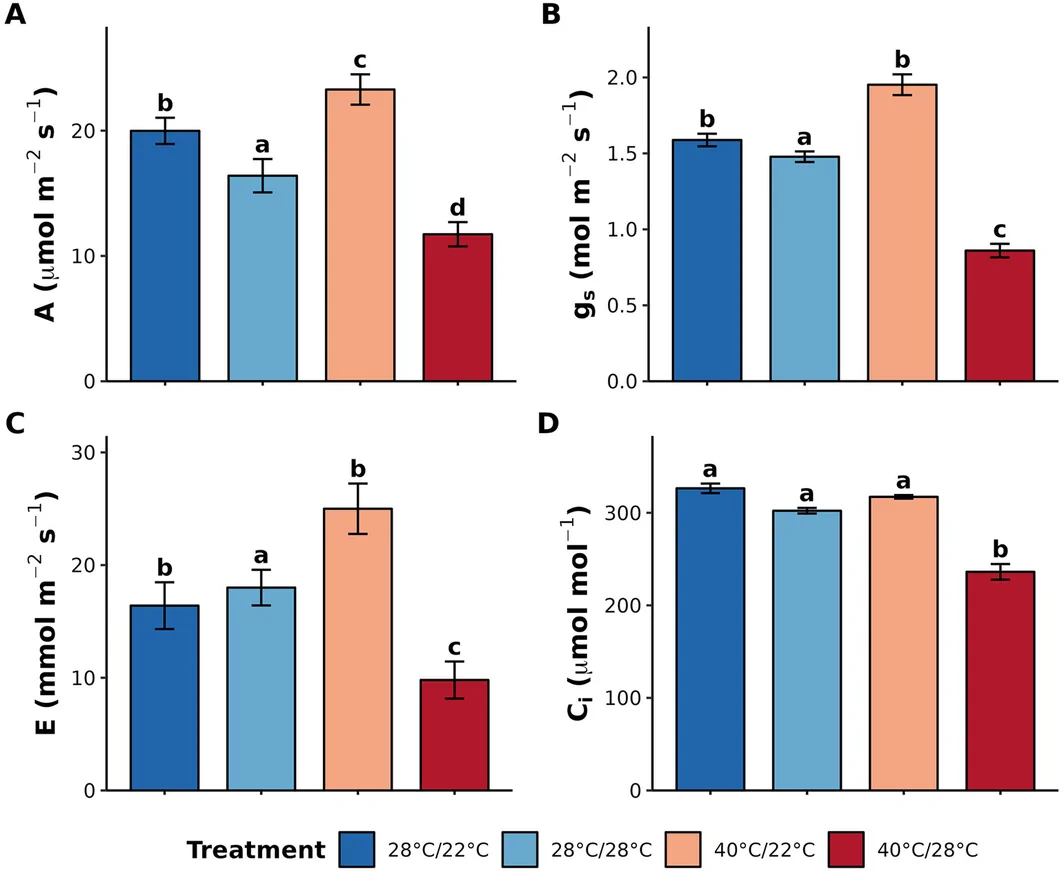
Leaf gas exchange parameters of soybean plants under different day/night temperature regimes: Net photosynthetic rate (A), stomatal conductance (*g*_*s*_), transpiration rate (E), and intercellular CO_2_ concentration (*C*_*i*_). Bars represent the mean ± standard deviation (*n* = 5). Different lowercase letters indicate significant differences among treatments within each panel according to Tukey's HSD test at *p <* 0.05.

Chlorophyll *a* fluorescence analysis provided further insight into the state of the photosynthetic apparatus (Figure 3). The potential quantum efficiency of PSII (Fv/Fm) remained statistically unchanged across all treatments, suggesting the absence of chronic photoinhibitory damage. However, parameters related to active photochemistry were significantly affected: the effective quantum efficiency of PSII (YII) was highest under 40°C/22°C and lowest under 40°C/28°C, mirroring the gas exchange results. In terms of photoprotection, a significant increase in non‐photochemical quenching (NPQ) was observed only under 40°C/28°C (Figure 3C).

**FIGURE 3:**
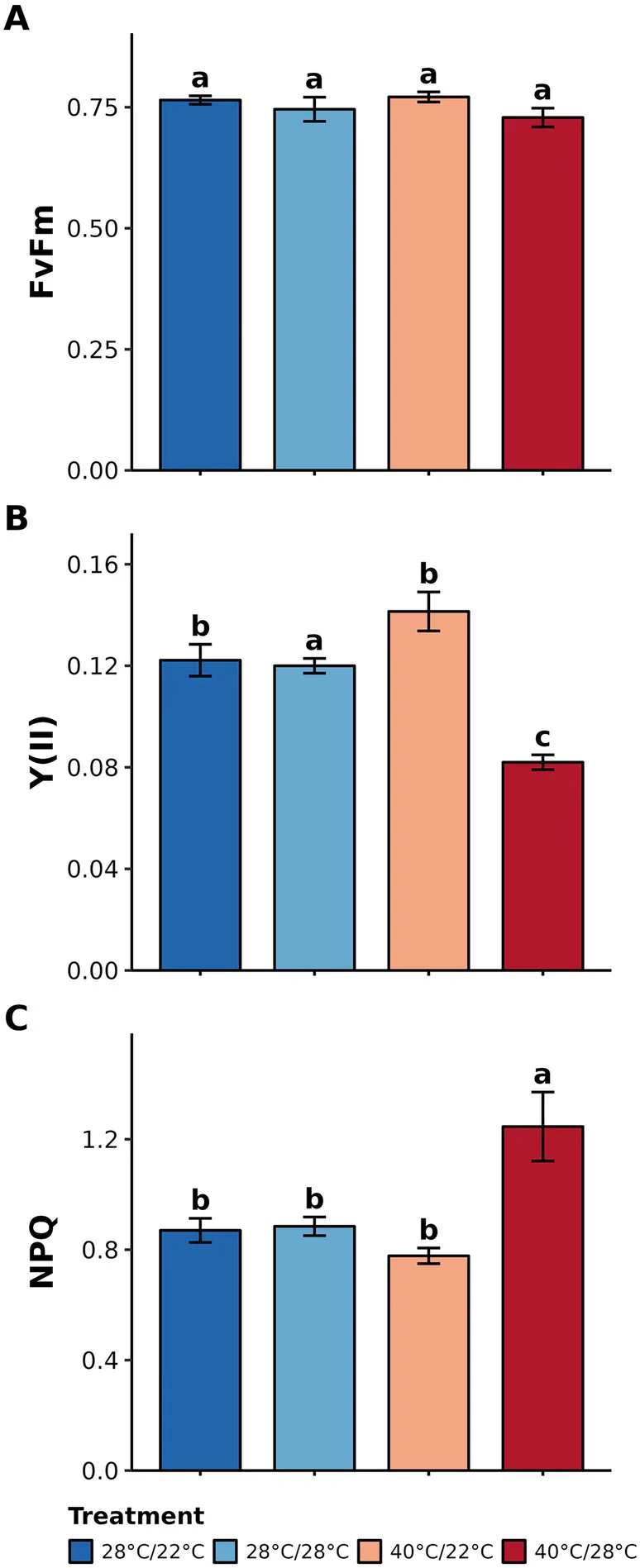
Chlorophyll *a* fluorescence parameters of soybean plants under different day/night temperature regimes: Potential quantum efficiency of PSII (Fv/Fm), effective quantum efficiency of PSII (YII), and non‐photochemical quenching (NPQ). Bars represent the mean ± standard deviation (*n* = 5). Different lowercase letters indicate significant differences among treatments within each panel according to Tukey's HSD test at *p <* 0.05.

### 3.3 Osmolyte Accumulation

To investigate the biochemical adjustments in response to heat stress, the accumulation of compatible osmolytes was quantified (Table 2). The responses varied among the compounds evaluated. Total soluble protein (TSP) remained similar to the control under high night temperature (28°C/28°C), increased under high day temperature (40°C/22°C), and dropped sharply under combined heat stress (40°C/28°C). Total soluble sugar (TSS) content peaked under high day temperature and was drastically reduced under combined heat stress. In contrast, proline and glycine betaine (GB) increased under heat stress, with proline rising strongly under high night temperature and reaching its maximum under combined heat stress. GB showed a pronounced increase under elevated night temperature and reached its highest concentration under combined heat stress (Table 2).

**TABLE 2:** Total soluble protein (TSP), total soluble sugar (TSS), proline, and glycinebetaine (GB) content in soybean leaves.

Variables	28°C/22°C	28°C/28°C	40°C/22°C	40°C/28°C
TSP (mg g^−1^ FM)	10.19 ± 0.52b	10.69 ± 1.08b	13.88 ± 1.54a	6.07 ± 1.09c
TSS (mg g^−1^ FM)	19.07 ± 0.83b	19.87 ± 0.90b	23.30 ± 2.40a	11.71 ± 1.13c
Proline (μg g^−1^ FM)	17.73 ± 0.65c	35.13 ± 2.19b	20.08 ± 0.56c	50.84 ± 1.76a
GB (μg g^−1^ FM)	1.97 ± 0.14d	5.12 ± 0.16b	3.92 ± 0.17c	10.73 ± 1.06a

*Note:* Data are represented as the mean ± standard deviation (*n* = 5). Different letters indicate significant differences among treatments according to Tukey's HSD test at *p <* 0.05.

### 3.4 Oxidative Stress Markers and Antioxidant Defense System

To determine the extent of oxidative damage induced by heat stress, the accumulation of H_2_O_2_ and lipid peroxidation (TBARS) was measured (Figure 4). Leaf H_2_O_2_ concentration increased in response to high night temperatures and peaked under combined heat stress (40°C/28°C), indicating elevated oxidative burden. The highest TBARS levels were also observed under combined heat stress, confirming severe oxidative damage to cell membranes. To counteract this challenge, soybean plants activated enzymatic antioxidant defenses in leaves, with responses varying among enzymes and treatments (Table 3). Superoxide dismutase (SOD) activity increased under high day temperature and reached its maximum under combined heat stress. Catalase (CAT) activity decreased under high night temperature alone (28°C/28°C) but increased under high day and combined heat stress. Ascorbate peroxidase (APX) activity was unchanged under high night temperature, increased under high day temperature, and peaked under combined heat stress. Thus, combined heat stress was the only treatment that maximized all three enzymes simultaneously, highlighting a coordinated enzymatic response to intensified oxidative stress (Table 3).

**FIGURE 4:**
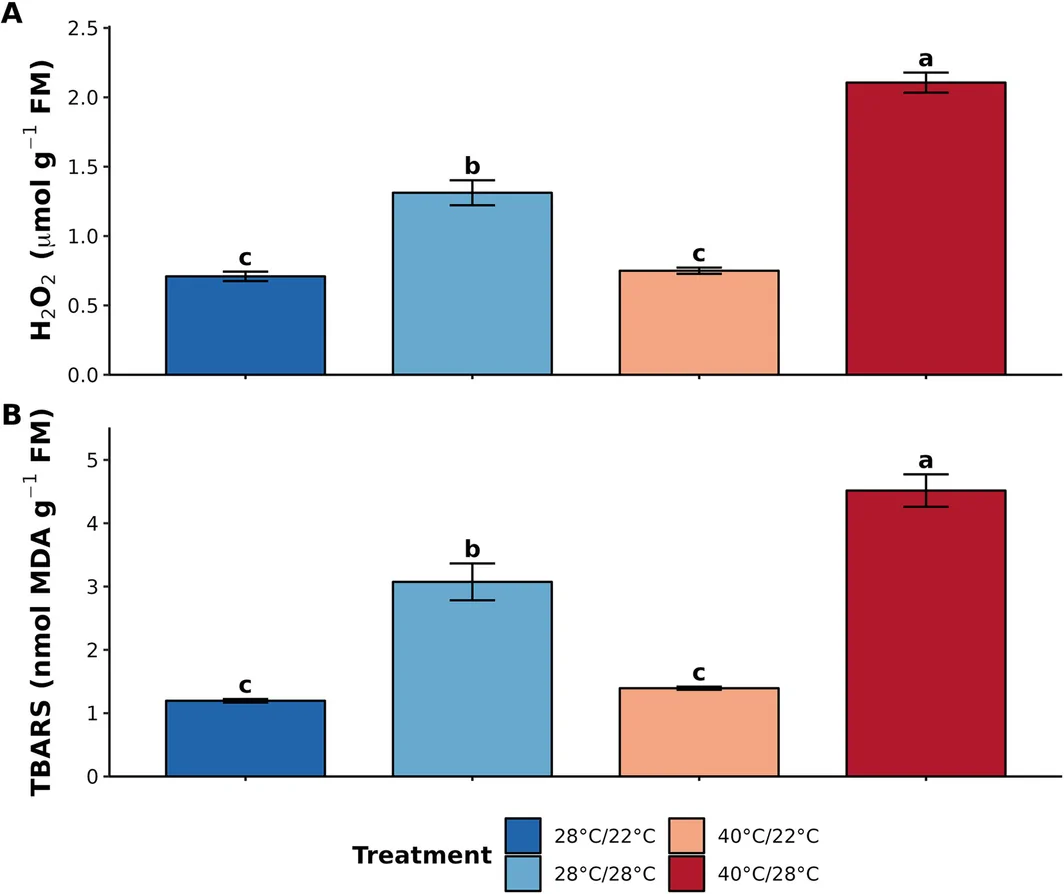
Oxidative stress markers in soybean leaves under different day/night temperature regimes: Hydrogen peroxide (H_2_O_2_) and lipid peroxidation (TBARS). Bars represent the mean ± standard deviation (*n* = 5). Different lowercase letters indicate significant differences among treatments within each panel according to Tukey's HSD test at *p <* 0.05.

**TABLE 3:** Activity of superoxide dismutase (SOD), catalase (CAT), and ascorbate peroxidase (APX) in soybean leaves.

Variables	28°C/22°C	28°C/28°C	40°C/22°C	40°C/28°C
SOD (U mg^−1^ protein)	0.92 ± 0.05c	0.82 ± 0.03c	1.78 ± 0.06b	2.30 ± 0.24a
CAT (U mg^−1^ protein)	2.87 ± 0.19b	1.75 ± 0.14c	3.68 ± 0.20a	3.93 ± 0.13a
APX (U mg^−1^ protein)	1.45 ± 0.11c	1.56 ± 0.19c	2.31 ± 0.29b	3.16 ± 0.38a

*Note:* Data are represented as the mean ± standard deviation (*n* = 5). Different lowercase letters within the same row indicate significant differences among treatments according to Tukey's HSD test at *p <* 0.05.

### 3.5 Integrated Analysis of Plant Responses

To provide an integrated overview of soybean responses to the thermal regimes, a principal component analysis (PCA) was performed (Figure 5). The first two principal components (PC1 and PC2) explained 82.1% of the total variance, indicating that this biplot captured most treatment‐related variation. A clear separation among treatments was observed mainly along PC1, defining a gradient of stress intensity. Control (28°C/22°C) and high daytime temperature (40°C/22°C) samples grouped on the positive side of PC1, reflecting similar overall physiological profiles. By contrast, high nighttime temperature (28°C/28°C) occupied an intermediate position, whereas combined day–night heat stress (40°C/28°C) was clearly separated on the negative side of PC1, forming the most distant cluster.

**FIGURE 5:**
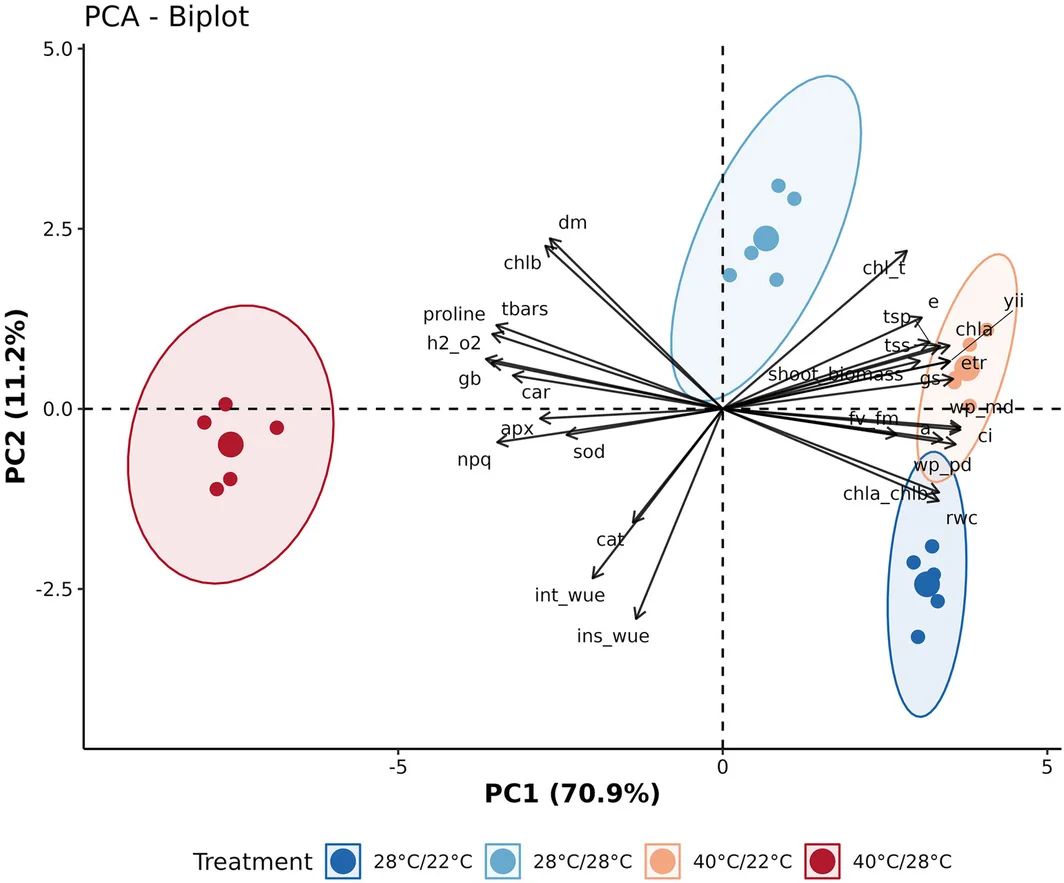
Principal component analysis (PCA) integrating growth, water status, gas exchange, photochemical, osmolyte, oxidative, and antioxidant variables of soybean plants under contrasting day/night temperature regimes. PC1 and PC2 explained 82.1% of the total variance and separated treatments according to stress intensity, with control (28°C/22°C) and high day temperature (40°C/22°C) grouped apart from high night (28°C/28°C) and combined heat stress (40°C/28°C): Variables: *a*, net assimilation rate; *apx*, ascorbate peroxidase; *car*, total carotenoids; *cat*, catalase; *chla*, chlorophyll *a*; *chla_chlb*, chlorophyll *a* to *b* ratio; *chlb*, chlorophyll *b*; *chlt*, total chlorophylls; *ci*, intercellular ${\mathrm{CO}}_{2}$ concentration; *dm*, membrane damage; *e*, transpiration rate; *etr*, electron transport rate; *fvfm*, maximum quantum yield of photosystem II; *gb*, glycine betaine; *gs*, stomatal conductance; $H_{2}O_{2}$, hydrogen peroxide; *ins_wue*, instantaneous water use efficiency; *int_wue*, intrinsic water use efficiency; *npq*, non‐photochemical quenching; *proline*, proline; *rwc*, relative water content; *shoot_biomass*, shoot biomass; *sod*, superoxide dismutase; *tbars*, thiobarbituric acid reactive substances; *tsp*, total soluble proteins; *wp_md*, midday leaf water potential; *wp_pd*, predawn leaf water potential; *yii*, effective quantum yield of photosystem II.

The ordination pattern was associated with two contrasting sets of traits. The control and 40°C/22°C groups were aligned with variables related to growth and photosynthetic performance, including shoot biomass, relative water content, net CO_2_ assimilation, stomatal conductance, transpiration, and PSII efficiency. In contrast, treatments including elevated nighttime temperature were associated with stress and defense markers, such as proline, glycine betaine, H_2_O_2_, TBARS, and antioxidant enzyme activities. Overall, this integrated analysis reinforces that elevated nighttime temperature is the main driver of physiological disruption in this experimental system, and that its combination with high daytime temperature amplifies stress damage while shifting metabolism toward defense.

## 4 Discussion

### 4.1 Impact of Asymmetric Warming on Growth and Water Status

This study demonstrates that soybean physiological and biochemical responses to elevated temperatures are strongly determined by the timing and interaction of thermal stress across the diurnal cycle. Consistent with climate projections indicating faster warming at night than during daytime maxima (Davy et al. 2016), our results reveal a clear asymmetry in plant response. While isolated high daytime temperature (40°C/22°C) enhanced gas exchange and photochemical performance, a pattern that can reflect short‐term plastic acclimation to daytime heat (Djanaguiraman et al. 2013), the inclusion of high nighttime temperature (28°C/28°C and 40°C/28°C) triggered broader physiological impairments. This agrees with evidence that elevated nighttime temperature is often a major driver of carbon imbalance, yield penalties, and metabolic disruption in soybean and other crops (Kucharik and Serbin 2008; Yang et al. 2023). Moreover, the combined regime did not produce a merely additive effect, but rather a stronger collapse in water status and photosynthetic function, supporting the view that restricted nocturnal recovery is a key bottleneck under sustained heat exposure (Siebers et al. 2015; Yang et al. 2023).

Growth responses followed the same pattern of interaction. Isolated high day (40°C/22°C) and high night (28°C/28°C) treatments did not significantly reduce shoot biomass, whereas combined heat stress (40°C/28°C) caused a clear biomass decline. This contrasts with reports showing that high night temperature alone can reduce biomass through enhanced nocturnal respiratory carbon loss (Lin et al. 2021; Yang et al. 2023). Under our conditions, soybean plants appear to retain partial compensatory capacity during 20 days of isolated thermal stress, but this resilience was lost when high temperatures persisted across both day and night. This interpretation is consistent with studies showing that combined stressors shorten effective growth duration and reduce dry matter accumulation more severely than isolated factors (Li et al. 2024).

While most available literature focuses on the consequences of thermal stress during critical reproductive stages, such as flowering and seed filling, where high nighttime temperatures directly disrupt assimilate utilization, reduce grain weight, and trigger severe yield penalties (Yang et al. 2023; Sankarapillai et al. 2025), our findings bring novel evidence regarding early vegetative vulnerabilities. Specifically, we demonstrate that the physiological bottleneck under combined day–night heat is established much earlier during crop development (V2 stage). At this phase, before any reproductive structures are formed, the continuous thermal stress and the lack of a nocturnal cooling period are already sufficient to cause a significant decline in vegetative biomass accumulation. This indicates that the classic yield reductions observed at maturity in field studies (Ding et al. 2024; Sankarapillai et al. 2025) might be fundamentally underpinned by a cumulative, early‐stage disruption of key metabolic and physiological processes.

The biomass penalty under combined stress was closely associated with the deterioration of the plant water status. Elevated temperature increases vapor pressure deficit and transpirational demand (Nakano et al. 2015), but dehydration severity depends on the plant's ability to rehydrate overnight. In our experiment, pre‐dawn water potential (Ψ_*PD*_) declined markedly in treatments with high nighttime temperature, indicating incomplete nocturnal rehydration, and this was followed by very low midday water potential (Ψ_*MD*_) under combined stress. Together with reduced relative water content, these data indicate that nighttime heat compromised recovery between photoperiods and amplified daytime hydraulic strains.

Sustained dehydration under high temperature was accompanied by a strong increase in membrane damage, reaching approximately threefold the control level under 40°C/28°C. This result is physiologically coherent with heat‐induced destabilization of membrane lipids, which increases permeability and electrolyte leakage (Wahid et al. 2007). Similar structural impairment has been reported in soybean leaves exposed to heat stress (Djanaguiraman et al. 2011). Therefore, loss of membrane integrity likely acts as a central mechanism connecting hydraulic dysfunction with downstream metabolic impairment and reduced biomass accumulation under combined day–night warming.

### 4.2 Photosynthetic Responses: Stimulation Versus Inhibition

The photosynthetic apparatus displayed strong plasticity across thermal regimes. The increase in chlorophyll *a* under isolated high daytime temperature (40°C/22°C) suggests metabolic upregulation and enhanced light‐use capacity, a pattern often associated with thermotolerant phenotypes under transient daytime heat (Ding et al. 2024). By contrast, the accumulation of chlorophyll *b* and carotenoids under combined stress (40°C/28°C) is consistent with a protective adjustment. In this context, carotenoids contribute both to stabilization of thylakoid structure and to antioxidant buffering of reactive oxygen species generated under excess excitation pressure (Gill and Tuteja 2010).

Gas exchange responses revealed a clear contrast between isolated daytime warming and treatments including nighttime heat. High daytime temperature alone (40°C/22°C) increased net photosynthesis, stomatal conductance, and transpiration, together with higher soluble sugar content, indicating that soybean can temporarily exploit warmer daytime conditions when hydration and nocturnal recovery are maintained. Notably, this elevated stomatal opening under isolated daytime heat coexisted with a significant decline in leaf relative water content. This response reveals a critical physiological trade‐off where thermal avoidance via transpirational evaporative cooling takes precedence over strict leaf water conservation. To protect the thylakoid membrane integrity and photosynthetic machinery from severe thermal damage, the plant maintains open stomata to maximize heat dissipation. This intense transpirational flux creates a transient hydraulic deficit where high atmospheric evaporative demand temporarily outpaces root water transport capacity, causing a minor drop in tissue hydration despite optimal soil moisture. However, combined day–night heat (40°C/28°C) caused a pronounced collapse of these variables. Importantly, this decline occurred with a marked reduction in intercellular CO_2_ concentration (*C*_*i*_), indicating that photosynthetic inhibition was primarily driven by stomatal limitation rather than by direct biochemical impairment of carboxylation capacity. This interpretation agrees with the hydraulic constraint framework proposed for stressed soybean leaves, in which water‐status signals promote stomatal closure and secondarily reduce carbon fixation (Alves et al. 2024; Djanaguiraman et al. 2013).

Chlorophyll fluorescence further clarified the inhibitory mechanism. The stability of Fv/Fm across treatments indicates that chronic photodamage to PSII reaction centers was largely avoided. In contrast, the marked reduction in YII under combined heat paralleled the suppression of carbon assimilation, consistent with a regulated downshift of photochemistry when stomatal closure constrains CO_2_ availability. Under this condition, the significant rise in NPQ indicates enhanced thermal dissipation of excess excitation energy, a key photoprotective strategy that limits photooxidative damage when electron use by carbon metabolism is restricted (Lima Neto et al. 2017).

### 4.3 Metabolic Adjustments and Oxidative Stress

The effects of the thermal regimes on carbon metabolism provide biochemical support for the carbon hunger framework proposed for soybean under nighttime warming (Yang et al. 2023). The increase in total soluble sugars (TSS) under isolated high daytime temperature (40°C/22°C) is consistent with the stimulation of photosynthesis and indicates a transient surplus of photoassimilates. In contrast, the strong depletion of TSS under combined heat (40°C/28°C) reflects a dual physiological constraint. This sharp reduction in carbohydrate availability is heavily driven by the severe daily stomatal limitation observed during the day, which restricted carbon assimilation and limited the replenishment of the leaf carbohydrate pool (Djanaguiraman et al. 2013). This primary source limitation operates in conjunction with a hypothesized acceleration of dark respiration rates under elevated nighttime temperatures, which enhances the nocturnal consumption of remaining carbon reserves (Yang et al. 2023). Given that respiratory fluxes were not directly quantified in this study, this accelerated nighttime consumption represents a highly probable hypothesis that functions alongside the daytime photosynthetic impairment to deplete the overall carbohydrate pool under compound thermal stress.

To cope with this metabolic pressure, soybean activated osmoprotective pathways, as evidenced by the strong accumulation of proline and glycine betaine under elevated nighttime and combined heat regimes. This pattern is consistent with the recognized roles of these compounds in osmotic adjustment, stabilization of proteins and membranes, and mitigation of oxidative stress (Wahid et al. 2007). Notably, osmolyte accumulation was particularly associated with treatments including nighttime warming, suggesting that nocturnal heat is a key trigger of this response. At the same time, the drop in total soluble proteins under 40°C/28°C suggests a metabolic tradeoff, with nitrogen and carbon resources being reallocated from constitutive protein pools to stress‐related synthesis pathways (Gill and Tuteja 2010).

Regarding enzymatic antioxidant defense, only combined day–night heat consistently induced simultaneous increases in SOD, CAT, and APX activities. This coordinated upregulation indicates that antioxidant demand peaks when daytime and nighttime stress are continuous and recovery windows are reduced. However, despite this robust enzymatic activation, H_2_O_2_ and TBARS also reached maximum values under 40°C/28°C, indicating that oxidative production exceeded detoxification capacity and resulted in persistent cellular damage (Shaffique et al. 2024). Conversely, under the isolated high nighttime temperature treatment (28°C/28°C), a distinct operational imbalance occurred within the antioxidant defense network, characterized by a concurrent increase in H_2_O_2_ accumulation and a significant decrease in catalase activity. Catalase is highly susceptible to thermal inactivation and protein degradation under stressful conditions. At the same time, nocturnal warming stimulates mitochondrial respiration rates, accelerating the generation of reactive oxygen species that rapidly dismutate into H_2_O_2_. Because catalase activity was compromised by the high nocturnal thermal regime, the remaining enzymatic pool was insufficient to detoxify this increased oxidant load, creating a severe metabolic bottleneck that promoted subsequent cellular oxidative damage.

The PCA synthesis reinforces this interpretation. Plants under combined day–night heat formed a tight and clearly separated cluster, distinct from all other treatments, while high nighttime temperature alone occupied an intermediate stress position. This multivariate pattern supports the concept that compound thermal extremes produce distinct physiological states rather than simple additive responses, and highlights the need to explicitly include diurnal thermal timing in crop stress modeling and risk projections (Li et al. 2024). Taken together, these findings confirm the central hypothesis that nighttime warming operates as the primary and most destructive driver of physiological disruption in soybeans. While high daytime temperature alone can be partly accommodated through functional plasticity, the high standalone impact of nocturnal warming on membrane integrity imposes a severe basal constraint that suppresses recovery capacity, causing cellular damage to culminate near its functional threshold under combined day–night stress.

## 5 Conclusions

This study confirms that the timing of thermal stress is a central determinant of soybean physiological resilience, with elevated nighttime temperatures being the dominant driver of physiological disruption rather than isolated daytime warming. While plants showed functional plasticity under high daytime temperature alone, the inclusion of high nighttime temperature impaired nocturnal recovery, intensified hydraulic dysfunction, and promoted carbon imbalance. As a result, combined day–night heat led to a cumulative exhaustion of photosynthetic performance near its functional threshold, accompanied by severe oxidative damage despite the activation of enzymatic antioxidant defenses. These findings indicate that breeding strategies and climate mitigation practices designed to improve soybean heat tolerance must explicitly target nighttime metabolism, nocturnal rehydration capacity, and recovery efficiency across the entire diurnal cycle.

## Author Contributions

**Gabriel Ferraz Guimarães** and **Jéssica de Jesus Santos:** formal analysis, investigation, methodology. **Rafael Vasconcelos Ribeiro:** writing – review and editing. **Milton Costa Lima Neto:** conceptualization, resources, supervision, writing – original draft, visualization, review and editing.

## Funding

This study was funded by Fundação de Amparo à Pesquisa do Estado de São Paulo (FAPESP) 2024/21237‐3. M.C.L.N. is sponsored by CNPq (301453/2022‐5). G.F.G. is sponsored by FAPESP 2025/20086‐4.

## Conflicts of Interest

The authors declare no conflicts of interest.

## Data Availability

The data that support the findings of this study are available on request from the corresponding author. The data are not publicly available due to privacy or ethical restrictions.
